# Accelerating entrepreneurship: Evidence from the incubation centers of management institutes of Dakshina Kannada

**DOI:** 10.1016/j.heliyon.2024.e34312

**Published:** 2024-07-09

**Authors:** Niyaz Panakaje, Arun A. Bhagwath, S.M. Riha Parvin, Madhura K, Ujwala Kambali

**Affiliations:** a YEN-REFINED, The Yenepoya Institute of Arts, Science, Commerce & Management (YIASCM), Yenepoya (Deemed to be University), Mangalore, 575018, India; b Institute of Management and Commerce, Srinivas University, Mangalore, India

**Keywords:** Incubators, Incubation centre, Entrepreneurship, Management institute, Students

## Abstract

**Purpose:**

Incubation centres are gaining significant prominence as they play a crucial role in providing supportive environment and encouraging entrepreneurship among students. Considering its importance, the study is intended to offer a thorough knowledge of incubators’ role in promoting entrepreneurship growth using data acquired from Dakshina Kannada Management Institutes. It also intends to investigate the efficacy and success of incubators using a variety of metrics.

**Methods:**

This study is based on the mixed-method approach, where specifically the concurrent qual-quant approach is adopted. An in-depth interview was undertaken, and a structured questionnaire was formulated to collect the data from final year Masters’ students from the Management Institutes. Qualitative analysis was narrated and quantitative analysis was carried out using SPSS 26 and AMOS 23, with descriptive statistics, *t*-test, regression analysis, and SEM which were utilized to evaluate the association between variables.

**Results:**

The study has highlighted the importance of reputation and credibility of the incubation centre, along with the significance of building a strong network as integral factors for learning from it. Overall, the study has found that students’ perception of education collaboration, and policy regulatory framework pertaining to incubation centres highly impacted the effectiveness of the incubation centre, which further enhances its success.

**Implications:**

This study helps to handle challenges for young entrepreneurs through proper direction and support, by refining their business ideas and collaborations with local and regional businesses to create a healthy entrepreneurship environment by fostering the integration between government bodies, financial institutions, and venture capitalists with the entrepreneurs and key stakeholders.

**Originality/rationale:**

As an exceptional highlight than other studies, this study focused on assessing the impact of awareness, perception, education and collaboration, policy and regulatory frameworks, and challenges on the effectiveness of incubators, and success of incubation centre. This has provided the answers to more comprehensive questions pertaining to incubation centre using mixed method approach. The combination of qualitative and quantitative results has identified novel findings which urges the incubation centres to identify the startups with high growth potential where the major challenges lie in convincing the students to implement their own business ideas.

## 1 Introduction

In India entrepreneurship has become a revolutionary force with 794 incubations and accelerators by having combined portfolio of 5.58K companies [1] that propels innovation, job creation, and economic growth in the current fast-paced global economy [2]. With their innovative concepts and flexible business models, start-ups are instrumental in reshaping sectors and expanding the realm of imagination [3]. The incubators for businesses play an important role in the growth of businesses that have grown so far [4]. A real approach that values inventions, taking risks, and adaptableness to start a business is what is considered as an entrepreneurship [5]. The businesses face challenges such as lack of funding, ties to the sector, and the requirement for mentoring, which stresses the need for aiding frameworks [6]. These startup incubations will entitle the entrepreneurs in different phases initiating from the selection of their business idea, pre-incubation, incubation, and post-incubation support to overcome these challenges. Incubators, which have conventionally been used to care for preterm infants, have amended in the business sectors to offer entrepreneurs a safe and promising atmosphere in their initial stage [7].

For the success of the early growing businesses, the incubators offer multiplicity of resources, coaching, trainings, workshops, networks [8,9] aiming at rapid development, in order to improve their likelihoods of success, and also contribution to the economy. Incubation models work as important facilitators for new businesses by providing access to market information, extending support in financial and legal aspects, and investment decisions [10,11]. Incubators, often affiliated with universities, research institutions, corporate organizations, or public agencies, emphasize on specific areas, industries, and services [7]. They basically provide physical workspace together with access to networks of industry experts and investors, assisting business development, training, and access to financial resources [12]. Therefore, because of the rapid technological advancements in this sector, incubations act as a driving force for the entrepreneurs for their great transformation in this digital age [13].

According to Karnataka states’ startup ranking 2021, it scored 68th percentile in the incubation support in reform area of 26 Action Points of the Startup Ranking Framework 2020 [14]. The educational institutes have played a major role in fostering entrepreneurship in the region of Dakshina kannada with its well-thought-out incubation initiatives [15,16]. These initiatives have made it easier for numerous businesses to grow across a range of sectors, from technology and healthcare to agriculture and renewable energy [17]. To enhance the opportunities for entrepreneurs within the incubator, the institutes have established partnerships with financial institutions, venture capital firms, and local and regional governmental organizations and these partnerships aim to foster the growth of entrepreneurship and ultimately support economic expansion [18,19].

The study has examined the concept of incubators and their significance in promoting entrepreneurship using information obtained from Dakshina Kannada Management Institutes. Due to the efforts made by the local management institutes to establish and administer effective incubation programmes, the district of Dakshina Kannada in the Southern Indian state of Karnataka has gained recognition for its strong start-up ecosystem. In this region, the major incubation set-ups are mainly concentrated on Information Technology (IT), medical, and engineering and also two incubations have come up with Micro, Small and Medium Enterprises (MSME) business incubations. These centres are providing good infrastructure with workspace, technological support and conduct of skill enhancement programs, workshops, trainings, networking, mentoring, connection with business experts, collaborations and funding facilities [20]. Despite the growing importance of incubation in promoting entrepreneurship, only a very few management students have registered in these incubations and there is limited research on their specific impact from the point of view of the regional context of Dakshina Kannada. Although there are 8 colleges which offer entrepreneurship education in Dakshina Kannada, this study helps to fulfil the gap between classroom-oriented entrepreneurship education and success of their business [21]. Although these educational institutions design and offer an extensive coursework with good curriculum on entrepreneurship, there is a need for understanding the efficacy of incubation centres in fostering successful startups [22]. In addition to examining the efficacy and success of incubators using a variety of metrics, this study attempts to present a thorough knowledge of the function that incubators play in hastening the growth of entrepreneurship and it expects a raise in their economic contribution to be around 17 % by 2030 [23]. This study specifically looks at the efficacy and success of incubation centres as reported by the students in an effort to evaluate their contribution in the acceleration of entrepreneurship.

## 2 Literature review

There is a growing recognition in the role which business incubators play in encouraging entrepreneurship as businesses benefit from their invaluable resources, networking opportunities, and mentorship. This literature review investigates the body of knowledge regarding the function of incubators in promoting entrepreneurship, using Dakshina Kannada's Management Institutes as a case study. The goal of this analysis is to provide a comprehensive understanding of how incubators promote innovation, entrepreneurial growth, local economic development. The collaborative, risk-taking, and experimental environments fostered by incubators are critical for the success of entrepreneurship.

For prospective business owners, particularly those who have completed their B-school education, incubators located within academic institutions serve as a priceless source of motivation. According to Ref. [9], these centres offer practical training, workshops, and coaching on a range of entrepreneurship-related issues, creating a nurturing environment that is conducive for the creation of ideas and growth of the firms. Incubators have an impact on the development of start-ups by providing essential components like finance access, networking opportunities, collaborative workspaces, and mentorship programmes.

The study conducted by Ref. [24] provides insight into the opinions of the businesses residing in government-funded incubators situated in Indian colleges concerning the calibre and scope of services offered and also draws an attention to how some services should be provided more effectively to make the most of the incubator's capabilities. Moreover [25], in his study conducted a gap analysis between tech-savvy entrepreneurs and creative entrepreneurs using qualitative approach. This gap impacts the effectiveness of incubation centre. Another study by Ref. [26] challenged the notion that company incubation is beneficial to all early-stage entrepreneurs as they provide a typology to illustrate that for certain entrepreneurs. Based on qualitative data from interviews with firm owners and managing directors of ten incubators, incubation may not be beneficial or even desired. Apart from the qualitative studies in this domain [9], claim that the effect of incubation centres in inspiring start-up entrepreneurs can be achieved through skill development, networking opportunities, fostering innovations, and entrepreneurial mentality using quantitative research with 271 graduates from business school. However, this study only identified the components of the role of incubation centre, but failed to test its relationship with entrepreneurship development. Moreover, in the context of USA [27], conducted a review to assess the relevance of incubation centres in enhancing the early-stage entrepreneurial paths, which again explored the importance of networking, funding and mentorship. However, general studies on numerous factors influencing student's entrepreneurial intentions have found the relevance of business incubation centres [28]. Furthermore [29], conducted qualitative research to assess the effectiveness of Brazilian university-based technology incubators in facilitating the transfer of academic research into new businesses, and discovered an absence of focus on subsidiary development despite a preference for projects with high university collaboration potential, demonstrating underutilization of this channel for research transfer. Furthermore [30], explored important performance metrics, support systems, and entrepreneurial hurdles using quantitative analysis and qualitative case studies from Global South, demonstrating links between Technology incubation centre success and variables such as mentorship and financial access. In line with this [31], conducted a study to assess the impact of University Business Incubation (UBI) on the entrepreneurial performance of Nigerian university students by employing a questionnaire-based approach. The results indicated that business incubation had a significant impact on students' entrepreneurial performance. Further [32], claims that focusing on entrepreneurship education in institutions has favourable results, like favourable electronic word-of-mouth, because it's seen as an inventive way to draw in more students.

Policies and programmes were started in India [19] to encourage student entrepreneurship. Strategies at each stage require emphasis, and student perceptions vary, indicating the need for promoting the growth of entrepreneurship and ultimately contributing to economic growth. Business incubators encounter various obstacles in their efforts to promote entrepreneurs [33]. identified a few of these issues as lack of sponsorship, production space, modern technology facilities, and development into new areas. According to Refs. [34,35], theories that provide perspectives on the processes have an impact on the function that business incubators play as catalysts for entrepreneurship as the institutional theory is concerned with how organizations become legitimate by conforming to the norms, values, and customs of their environment [36] and provides a solid framework for understanding how business incubators work as entrepreneurship accelerators. By demonstrating how incubators promote start-up success, innovation, and economic growth, they offer a rich context for analysing the operations and outcomes of incubation programmes.

Numerous studies reveal extensive research in the domain of incubation centres, demonstrating the significance of the incubation centre. However, majority of the studies have employed qualitative studies with interviews and case studies which may have low generalizability. At the same time, some quantitative studies have also claimed the significance of incubation centres. However, very rarely studies have implemented mixed method approach and provided an extensive overview of incubation centre in accelerating entrepreneurship development from the perspective of students. There is a scarcity of tested research on the role of incubation centres in entrepreneurship development. This study by focusing on awareness, perception, education and collaboration, policy and regulatory frameworks, the role of incubators, challenges, and success of incubation centre, will provide answers for the more comprehensive questions pertaining to incubation centre.

As a whole, the existing research acknowledges the importance of incubators and their role in supporting entrepreneurial aspirations, but it mostly ignores the obstacles and limitations that limit their efficacy, and there is also a dearth of thorough research on the integration of entrepreneurship education into academic programmes and its impact on students’ attitudes and aspirations for launching their firms. The impact and efficacy of incubation centres should therefore be investigated to get past roadblocks and fully grasp the potential of business incubators as institutional entrepreneurship boosters.

## 3 Theoretical framework and hypothesis development

As stated by Albert Bandura's Social Cognitive Theory, individuals acquire knowledge by seeing the actions and results of others. Students' knowledge of resources and success stories in an incubation centre affect their engagement, motivation, and resource usage. It focuses on self-efficacy, the idea that confidence is influenced by the accomplishments of others and observational learning, in which students emulate successful businesses. Along with highlighting goal-setting and motivation, it demonstrates how resource knowledge propels entrepreneurial endeavours [37,38].

Understanding and interpreting information in one's environment is the focus of perception theory. Students' opinion about an incubation centre's standing, the calibre of resources, support networks, and the general environment are crucial in influencing the extent to which they are and the frequency they use the centre's offerings. Although negative impressions can deter students from taking full advantage of the centre's resources, positive attitudes frequently lead to increased involvement and utilization [24].

The institutional theory primarily examines how formal and informal institutions affect the actions and outcomes of organizations. A centre for incubation's ability to attract participants and manage its operations can significantly impact its financial situation, legislative policies, and institutional support network. According to Refs. [39,40], and [41], regulatory compliance, funding opportunities, and governmental activities can all help to increase the incubation center's sustainability and efficacy.

The study of resilience theory examines how people and organizations deal with adversity and go beyond barriers to survive and the success of an incubation centre can be measured by how well it can negotiate and get over challenges including scarce resources, unstable market conditions, and financial constraints [[42], [43], [44]]. The incubation centre must be built resiliently, a flexible culture must be fostered, and entrepreneurs must be given the tools they need to overcome challenges, if it is to be sustainable and successful in the long run.

Thus, these theories emphasize the importance of self-efficacy and observational learning, the importance of student opinions, the power of institutions, and the capacity to overcome obstacles. Incubation centres can enhance their support towards business ventures facing challenges by implementing concepts from these theories.

Based on the theoretical framework the following hypotheses are developed.

### 3.1 Student awareness

In an incubator context, students' awareness is crucial in deciding their level of engagement and how they use the centre's resources, as emphasised by the Social Cognitive Theory. Individuals learn by observing others, and students are inspired to engage in the centre's activities when they witness their classmates or successful business owners using its resources [45] with the positive role modelling, encouraging students to emulate this attitude, which strengthens it even more. Additionally, social contact in the centre promotes awareness and involvement by providing opportunities for experience sharing and information acquisition from others [38] and by fostering a supportive environment that encourages involvement and the effective use of resources. It often highlights the importance of observational learning and the part that student awareness plays in encouraging interaction with the incubation centre [46] in order to promote students' engagement with the incubation centre, the theory of social cognition generally highlights how important it is for students to be aware of it. Additionally, it highlights how crucial social interaction and observational learning are to the centre's capacity to support entrepreneurial ventures. Therefore, it is hypothesised that;

H1 Student awareness significantly impacts the effectiveness of the incubation centre.

### 3.2 Student perception

The perception theory examines the thought processes by which individuals interpret their environment and generate beliefs, dispositions, and choices and also highlights the value of student viewpoints in determining the centre's effectiveness within the framework of an incubation centre [24,47]. Students' opinions span a wide range of subjects, including the centre's reputation, services, and support systems, based on their personal experiences and relationships with others. According to Refs. [38,48], there is an increased degree of student involvement and utilization of the centre's services due to their good attitudes and faith in its abilities and negative viewpoints may discourage involvement and lessen the effectiveness of the centre. The key to optimizing the centre's impact, encouraging positive involvement, and promoting entrepreneurial success is managing and influencing perceptions through efficient communication and service delivery. Hence, it can be hypothesised that;

H2 Student perception significantly influences the effectiveness of incubation centre.

### 3.3 Education along with collaboration

The institutional theory clarifies how formal and informal institutions influence the conduct and results of organizations. Under the proposed framework, cooperation and education play a major role in an incubation center's overall efficacy. According to Institutional Theory, the provision of educational resources and opportunities by the centre enables it to conform to prevalent institutional norms, hence augmenting its legitimacy and efficacy [39,40]. Education is considered an institutional norm in the entrepreneurial environment. Establishment of legitimacy and credibility within the ecosystem are achieved by incubation centres through the promotion of collaboration, another institutional norm. To maximize its impact on fostering entrepreneurship, the centre can achieve institutional isomorphism with prevalent norms, support entrepreneurial initiatives more effectively, and achieve this through enabling collaboration and partnerships [41,49]. Hence, it can be hypothesised that;

H3 Education along with collaboration remarkably impacts the effectiveness of incubation centre.

### 3.4 Policy and regulatory framework

The center's operational and goal-achieving capacity is greatly impacted by the policies and regulatory framework that surround it, by institutional theory. According to Refs. [41,49], compliance with rules and regulations that support them can enhance effectiveness by streamlining operations, promoting innovation, and providing access to resources. Yet, bureaucratic or legal barriers could make things harder and prevent funding opportunities and stakeholders participation. Understanding and navigating the larger institutional framework is critical in enhancing the center's ability to support entrepreneurial pursuits, as per institutional theory [[39], [40], [41]]. By endorsing regulatory reforms and aligning with policies that promote its objectives, the center can improve its capacity to surmount external constraints and minimize barriers.

H4 Policy and regulatory framework significantly influence the effectiveness of incubation centre.

### 3.5 Incubation Centre's success

The aforementioned theory underscores the importance of the incubation centre's effectiveness in facilitating business pursuits; its efficacy is determined by its ability to adjust and evolve when confronted with novel challenges, in addition to its initial resources and capabilities, in accordance to the resilience hypothesis [44]. To enhance the likelihood of effectively supporting entrepreneurial aspirations, an incubation centre must possess the capacity to maintain its core functions, adapt its strategies, and implement novel concepts in reaction to changing circumstances [50]. By increasing resilience, the centre is better able to withstand setbacks and continue assisting firms despite difficulties. Therefore;

H5 Effectiveness of incubation centre significantly impacts their success.

### 3.6 Challenges faced by the incubation centre

It acknowledges that challenges directly affect how well an incubation centre performs. According to resilience theory, a centre's resilience and, eventually, success are greatly influenced by how it handles obstacles like financial restraints, legal restrictions, or resource shortages. An incubation centre that is resilient, views setbacks as chances for improvement, looking for ways to go past them by forming alliances, looking for creative solutions, and using other resources [43,51]. The centre can improve its ability to support entrepreneurs, boost its resilience, and promote entrepreneurial endeavours more successfully overall by handling and navigating problems with skill. Hence, it is postulated that;

H6 Challenges faced have significant impact on incubation centre's success.

## 4 Conceptual model

**Fig. 1:**
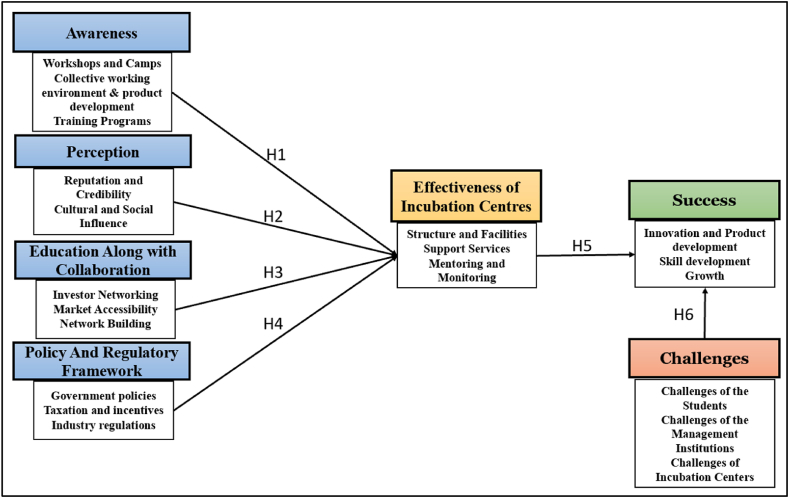
Conceptual model.

Based on the hypothetical relationship discussed earlier, the following conceptual model has been framed (See Fig. 1)

Developed by the Researcher

## 5 Materials and methods

This study is based on the mixed method approach, where the concurrent qual-quant approach is adopted. This mixed method research is a combination of quantitative and qualitative elements to gain a broad notion of a particular problem [52]. Mixed method approach is applied in the study to provide a comprehensive understanding of students on incubation centre through structured questionnaire which was built based on the existing literature and also to explore their general view on incubation centre through open ended questions. This method helps to evaluate the similarity and differences in student views, thereby provides effective factors necessary for successful incubation centres through integrated analysis.

### 5.1 Step 1: Research question/hypothesis formulation

Considering the relevance of incubation centres in entrepreneurship development and scarcity of studies in this area, particularly focusing on student's views, an attempt has been made to address two important questions from the perspective of Management students i.e. What is the role of incubation centres in accelerating the development of entrepreneurship? and how effective and successful are the incubation centres according to students? Firstly, a broad conceptual notion of understanding the incubation centre from students' perspectives were built upon the existing literature and observation, based on which qualitative and quantitative inventories were constructed, which included the major variables as awareness, perception, education and collaboration, the role of incubators, challenges, and success of incubation centre. These constructs were identified through the proper theoretical background and hypotheses were further formulated to implement quantitative analysis by keeping the research question into focus.

### 5.2 Step 2: Methodological selection

Further, the research was implemented using concurrent qual-quant approach by undertaking qualitative and quantitative study simultaneously without mixing the observation and results of both the approaches to explore a comprehensive, unknown and novel findings to dig the reality and feasibility of incubation centre operations according to the students. In concurrent mixed analysis, researcher can evaluate several types of data i.e. qualitative, quantitative or mixed data simultaneously, which means they may examine data from the same study using both quantitative and qualitative methodologies at the same time [53]. Considering its relevance, present study has implemented this approach which is further described below.

### 5.3 Step 3: Data collection

After deciding the methodology to suit our research direction, qualitative and quantitative data collection was executed by giving due importance to ethical consideration at two different settings.

**Ethical Consideration:** The questionnaire used in this study was approved by the Scientific Research Board (SRB/124/23) of YIASCM, Yenepoya (Deemed to be University), Mangaluru in accordance with the university's research policy and procedures on 25th May 2023. Moreover, oral consent was taken from the students before the in-depth interview and questionnaire distribution. All the collected responses pertaining to personal identifiable details of the respondents were kept confidential by the researcher.

**Qualitative Data Collection:** Here, firstly, 8 final year MBA and 4 final year M. Com students were approached, which reached saturation considering their inclusivity with the incubation centre from three universities of Dakshina Kannada district. 4 students were from Private University, 5 students were from Government University and remaining 3 from Deemed to be University. In order to include the heterogeneity of participants, purposive sampling was used. An in-depth interview on the above-mentioned constructs was taken, which was later transcribed by the researchers.

**Quantitative Data Collection:** Structured questionnaire was formulated for the above-mentioned constructs considering the various previous literature and observations, which was further validated through content and construct (convergent and discriminant) validity along with a reliability check. The statements were measured using five-point likert scale “Strongly agree” to “Strongly Disagree”. All the selected constructs proved to be valid with ASV being greater than MSV and Cronbach alpha above 0.7. After the pilot study, a sample size of 396 students was collected from the final year students of MBA and M. Com from various universities having management institutes based on simple random sampling.

### 5.4 Step 4: Data analysis

Following the data collection, analysis was carried out for both qualitative and quantitative study at two different settings as supported by Ref. [53].

**Qualitative Analysis:** An in-depth interview was recorded and narrated based on the in-depth notion given by the respondents. The first question was “What do you know about the incubation centre?”, “How are you aware about the incubation centres?”, “How do you perceive the incubation centre?”, “What is the role of education and collaboration with incubation centre?”, “How does the policy and regulatory framework affect the centre?”, “What is the role of an incubator?”, “What are the major challenges for incubators?”, “How do you think an incubation centre can be successful?” A few additional questions were also asked on this, to enhance the quality of the response. Further, to certify the confirmability of the data, audio recordings, and notes were kept by the researchers.

**Quantitative Analysis:** Quantitative analysis was conducted using SPSS 26 and AMOS 23, where descriptive statistics, *t*-test, regression analysis and SEM were used to assess the relationship between each variable.

### 5.5 Step 5: Integrated analysis

As a major part of the mixed method approach, the results of both quantitative and qualitative analysis were compared and contrasted as it provided in-depth inferences and suggestions for the existing problem [54,55]. Mixed research started combining quantitative (QUANT) and qualitative (QUAL) components in the 1980s. At the moment, mixed methodology (MM) has the greatest potential for accomplishing successful integration of the research process [56], whereby both techniques aid in a more thorough comprehension of the issue [57]. Considering which, present study implemented integrated analysis of both qualitative and quantitative results to compare and contrast students views and provide a comprehensive understanding of the problem.

The above steps were implemented while carrying out the research which was later reported sequentially as per the format of research article. Beginning with the introduction aiming to provide primer to the study, paper delves into existing research, theoretical framework and formulation of hypothesis. Following the hypothesis development, paper depicts conceptual model, describes research design, reporting results and discussion and finally concludes with the implication, future research and limitations.

## 6 Results and discussion

### 6.1 Sample characteristics

Out of 396 samples, 200 (50.5 %) students were female and the majority (73.7 %, N = 292) of the sample belonged to the management stream. Moreover, 128 students specialized in finance, and 84 students each specialized in logistics, port and supply chain management and dual specialization. Further, 88.9 % of students reported that they have entrepreneurship as one of their subjects in their course.

### 6.2 Data description and validation

#### 6.2.1 Awareness

Firstly, an attempt was made to understand their awareness, where they reported that workshops and camps helped them to understand how business incubators support start-ups (M = 4.1616, SD = 0.72156). Further, they indicated a high level of awareness about the collective working environment & product development in the incubation centre encouraging more business ideas through brainstorming (M = 4.2424, SD = 0.75427). Respondents also stated that content covering in training programs can enhance their understanding of how incubators can accelerate entrepreneurship (M = 4.3131, SD = 0.76170).

#### 6.2.2 Perception

Students highly perceived that the reputation and credibility of the incubation centre influence their confidence in the quality of services provided (M = 4.0000, SD = 0.84194). Further, they reported that their values of social circle, judge the impact of incubation centres on fostering innovation and entrepreneurship (M = 3.7778, SD = 0.92838).

#### 6.2.3 Education along with Collaboration

Further, the students were asked to provide their views on education along with collaboration. They stated that collaborative interactions of the incubation centre with the investors contribute to a more comprehensive understanding of entrepreneurial concepts (M = 4.0707, SD = 0.74293). Also, investor networking contributes to a more practical and applied understanding of entrepreneurship (M = 4.0707, SD = 0.78276). Moreover, learning from the incubation centre about market dynamics gives a more practical understanding (M = 4.1111, SD = 0.70999) and building a strong network, which are essential aspects of learning experience (M = 4.2828, SD = 0.78001).

#### 6.2.4 Policy and Regulatory Framework

Students highly agreed that clear government policies attract investments in incubator programs (M = 4.0707, SD = 0.84497). Moreover, they also agreed that taxation policies favour start-ups in their growth within the incubation ecosystem (M = 4.0101, SD = 0.82364). Students also believe that the incubation centre encourages experimentation and flexibility (M = 4.1515, SD = 0.75793) fosters entrepreneurship, and contributes to its effectiveness in nurturing start-ups (M = 4.1515, SD = 0.77117).

#### 6.2.5 Incubation Centres

The students view on the physical layout, support services, mentoring and monitoring of the incubation were also collected. The results indicated that a well-structured incubator layout and resources create an inspiring setting for start-ups to thrive (M = 4.1313, SD = 0.82550). Moreover, they agreed that the capital from angel investors, government organizations, economic development coalitions, venture capitalists, and others encourage entrepreneurs (M = 4.0303, SD = 0.81074). Students also reported that ongoing monitoring and feedback loops, contribute to the continuous improvement and growth among start-ups (M = 4.1313 SD = 0.80059).

#### 6.2.6 Challenges

Students’ views were also collected on the various challenges of the students, management institutions and incubation centres with regard to accelerating entrepreneurship. They strongly viewed that initial market entry barriers and competition are the major challenges a student may face (M = 4.0707, SD = 0.83290). Management institutes are facing challenges in providing a conducive and innovative environment for start-ups (M = 3.9798, SD = 0.87705) as per the students. Moreover, they have stated that incubation centres are facing a major challenge in identifying start-ups with high growth potential (M = 3.9697, SD = 0.89391).

#### 6.2.7 Success

Lastly, students provided their agreement on how the incubation centre can be successful by incorporating innovation, product development, and skill development. Students have reported that an incubator's shared resources can aid product development (M = 4.0707, SD = 0.95734) and enhance skill development leading to improved problem-solving within start-ups (M = 4.0808, SD = 0.87354). They have also recommended engaging alumni and successful graduates as mentors and advisors in the incubation centre (M = 4.1212, SD = 0.82090).

### 6.3 Stream/gender of the students and their view on incubation centre

The independent sample *t*-test reported the student's view on incubation centre with regard to Awareness, Perception, Education along with Collaboration, Policy and Regulatory Framework, Incubation Centres, and Success, which were found to be similar between commerce (M = 4.046, SD = 0.611) and management streams (M = 4.023, SD = 0.599) with the p-value above 0.05. Moreover, the gender showed no significant difference between males (M = 4.007, SD = 0.578) and females (M = 4.051, SD = 0.626) with respect to their view on incubation centre.

### 6.4 Role of incubation centre on its success using structural equation modelling

In order to test the structural equation modelling, checking its measurement model, reliability, validity and goodness of fit is essential. The initial stage was to confirm that the observed variables were related to the factors using hypothesis testing, followed by confirming the reliability and validity of these constructs (factors). A measuring model was created and evaluated for this aim using CFA. CFA was used to examine the hypothesised link between the observed variables and their constructs (factors), as well as to establish the constructs' reliability and validation. All the factor loading in CFA found to be above 0.5. Later, fit indices were generated to assess the model's overall fit. As per Hair et al. [58,59], Hu and Bentler [60], and Byrne [61,62] criteria, results reported a satisfactory goodness of fit for the model (χ^2^⁄df = 1.896; GFI = 0.914; AGFI = 0.911; CFI = 0.928; NFI = 0.909; RMSEA = 0.044). Furthermore, the results of reliability, validity, correlation along with mean and standard deviation are presented in Table 1.

**Table 1:** Correlation, reliability and construct validity.

	Items	Alpha	Mean	S.D.	Composite Reliability	AVE	Square Root of AVE	MSV	ASV	Awareness	Perception	Education Along with Collaboration	Policy And Regulatory Framework	Incubation Centres	Success
AWR	9	0.890	4.158	0.5348	0.893	0.561	0.749	0.555	0.531	**0.749**		–	–	–	–
PRC	10	0.798	3.815	0.6266	0.899	0.572	0.756	0.560	0.546	0.648^a^	**0.756**	–	–	–	–
ECL	10	0.907	4.103	0.5884	0.893	0.616	0.785	0.586	0.571	0.688^a^	0.675^a^	**0.785**	–	–	–
RF	10	0.808	4.028	0.5976	0.981	0.798	0.893	0.606	0.585	0.603^a^	0.573^a^	0.721^a^	**0.893**	–	–
EIC	16	0.879	4.034	0.6142	0.914	0.656	0.810	0.627	0.600	0.650^a^	0.690^a^	0.774^a^	0.807^a^	**0.810**	–
SUC	11	0.768	4.034	0.6609	0.960	0.766	0.875	0.647	0.607	0.605^a^	0.617^a^	0.737^a^	0.652^a^	0.744^a^	**0.875**

Note: EIC = Effectiveness of Incubation Centre; AWR = Awareness; ECL = Education along with Collaboration; SUC= Success; RF= Regulatory Framework; PRC= Perception.

a Correlation is significant at the 0.01 level (2-tailed).

The constructs Awareness (M = 4.158, SD = 0.5348), Perception (M = 3.815, SD = 0.6266), Education Along with Collaboration (M = 4.103, SD = 0.5884), Policy and Regulatory Framework (M = 4.028, SD = 0.5976), Incubation Centres (M = 4.034, SD = 0.6142), and Success (M = 4.034, SD = 0.6609) all of them showed above-average views of students with a good correlation between study variables with r exceeding 0.5 (P < 0.01**). As far as awareness among the students is concerned, it is highly correlated with education along with collaboration. It exhibits that an increase in education along with collaboration increases their awareness (r = 0.688**), policy and regulatory framework (r = 0.721**), and the effectiveness of the incubation centre (r = 0.774**) as well as its success (r = 0.737**). This shows the relevance of education along with collaboration and it acts as a major catalyst to enhance the effectiveness of the incubation centre.

The composite reliability of the constructs was 0.893 for Awareness, 0.899 for Perception, 0.893 for Education along with Collaboration, 0.981 for Regulatory Framework, 0.914 for Effectiveness of Incubation Centre and 0.960 for Success (Table 1). This is over the permissible threshold of 0.7. Moreover, Construct validity is the degree to which a collection of measured variables accurately represents the underlying construct they intend to assess [58]. In this study, construct validity was established by determining convergent validity, and discriminant validity. Convergent validity was examined by looking at the constructs' Average Variance Extracted (AVE). All indicators exhibited Average Variance Extracted above 0.5 (Table 1), which further proved the convergent validity of the constructs. According to Ref. [63] discriminant validity may be tested by comparing AVE's square root to the appropriate interconstruct squared correlation estimations. Table 1 provides an explanation for construct validity. The square root of the AVE values for all components was larger than the inter-construct correlations, indicating that the constructs had discriminant validity (The square roots of AVE for each construct were higher than the inter-construct correlations). Furthermore, it can be shown that the maximum and average shared variance of all six constructs were smaller than AVE. Thus, the measuring model demonstrated strong construct validity and favourable psychometric features. The study also used a multicollinearity test to assess the instruments' reliability. According to Ref. [64], the Variance Inflation Factor (VIF) statistics, which is a measure of collinearity, were found to be below the threshold of 5. This indicates that the independent and dependent variables demonstrate appropriate level of correlation. The resultant number validates the suggested model's quality of fit. Thus, structural equation modeling may be used to examine the hypothesis that has been supported by data by illustrating how one variable affects another.

**Fig. 2:**
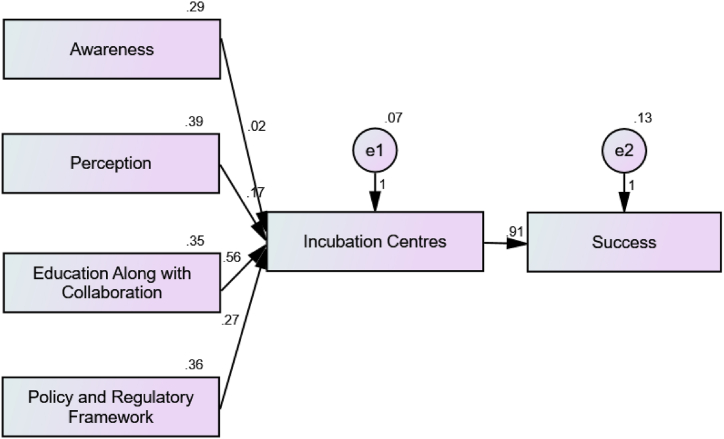
Structural Equation Modelling on the Role of Incubation Centre on its Success.

Structural equation modelling was used to test the conceptual model. It was reported that education along with collaboration highly contributed to the incubation centres (E = 0.557, P < 0.001) (H1). Moreover, regulatory framework (E = 0.270, P < 0.001) (H3) and students' perception (E = 0.167, P < 0.001) (H1) also significantly explained the role of the incubation centre. However, awareness of the incubation centre (E = 0.016, P = 0.530) showed no impact on the role of the incubation centre. Therefore, H4 is rejected. Further, the effectiveness of the incubation centre has immensely contributed to its success (E = 0.768, P < 0.001) (H5). Overall, it was illustrated from Fig. 2 and Table 2 that students’ perception, education, collaboration, and policy regulatory framework highly impacted the incubation centre, which further enhanced its success according to students.

**Table 2:** Role of the Incubation Centre on its Success.

Hypothesis	Relationship			Std. Est.	Un. Est.	S.E.	C.R.	P	Inference
H1	EIC	<---	PRC	0.224	0.167	0.022	7.663	***	Highly Significant
H2	EIC	<---	ECL	0.702	0.557	0.023	23.999	***	Highly Significant
H3	EIC	<---	RF	0.346	0.270	0.023	11.816	***	Highly Significant
H4	EIC	<---	AWR	0.018	0.016	0.026	0.628	0.530	Not Significant
H5	SUC	<---	EIC	0.768	0.908	0.038	23.808	***	Highly Significant

Note: EIC = Effectiveness of Incubation Centre; AWR = Awareness; ECL = Education along with Collaboration; SUC= Success; RF= Regulatory Framework; PRC= Perception.

### 6.5 Challenges of incubation centre

Moreover, H6 hypothesize that Challenges faced has significant impact on incubation centre's success. To test this hypothesis, multiple regression analysis was implemented which checked the effect of challenges in impacting the success of the incubation centre. As per the results, low level of perceived challenges does not reduce the influence of the incubation centre on its success (β = 0.852, P < 0.001**). When there is low level of challenges, the role of the incubation centre on its success is more but moderate (β = 0.743, P < 0.001**), and high level of challenges (β = 0.567, P < 0.001**) reduce the influence of the incubation centre on its success. Hence, H6 is accepted.

### 6.6 Qualitative analysis

Here, the participants are denoted with P as their respective numbers.

#### 6.6.1 Knowledge and awareness of the incubation centre

An in-depth interview with 12 students gave a broader notion on the topic which stated “It is a place to dwell in the field of their interest” “start an own business” “these centres give the guidance” “became aware about it after joining the master's course” (P1; P2; P3; P4) (P5; P6; P7; P8; P9). “Place where new business can be started (P2; P10; P11), “Place where the feasibility of new idea is examined” (P3; P4; P12). These views indicated that they are aware that this is the platform to start a new business in their specialized field, where their ideas can be checked for feasibility. Most of them became aware of the incubation centre after joining the master's course.

#### 6.6.2 Perception of incubation centre

As far as their perception of incubation centre is concerned, they have provided the following notion “Provides guidance, support and motivates …” (P1; P2; P3; P4; P5; P6; P7; P8; P9; P10; P11; P12), “Provides temporary license and opportunity for a new business” (P2). When they were asked about how they perceive incubation centre, most of them stated that incubation centres are meant to provide guidance, support, and motivation for their business ideas. One of the participants perceived that it is the place where the temporary license is made available to start a new business and provides an opportunity for business.

#### 6.6.3 Education and collaboration

Moreover, students were asked to provide their notion on education and collaboration where they have provided the following views - “Improves extracurricular activities …” (P1; P5; P6), “own ideas will get a reality …” (P2; P7; P8; P9; P10; P11), “Provides with funding ….” (P3), “Institute must take a step forward to collaborate …” (P4; P12). Their notion of education and collaboration indicates that collaboration increases the scope of extracurricular activities in their institution. Further, it provides a platform to transform their ideas into actual reality and also helps them to get funding. They also recommend institutions to take a step forward to collaborate with the incubation centres, companies, investors etc.

#### 6.6.4 Policy and Regulatory Framework

Students’ views on the policy and regulatory framework of the incubation centre provided a few glances - “The centre must be set up based on the certification given by the authority” (P1; P2; P3; P6) “The Government must provide support to this centre” (P3; P4; P6; P7; P11; P12) “not aware” “no comments …” (P5; P8; P9; P10). Here, the students stated the certification of incubation centre, at the same time they stated the importance of Government support in enhancing incubation centre and encouraging new businesses.

#### 6.6.5 Role of incubators

The in-depth interview on the role of incubators provided the following notions - “Visiting the institutions ….” (P1), “Promoting the incubation centre ….” (P2; P3; P4; P5; P6; P7; P8; P9), “Guide the newbies with their idea …” (P3; P10; P11), “Know about the changing market need ….” (P4; P1; P12). Students reported that the incubators must visit their institutions and promote their centre. Moreover, they shall guide the students with their ideas. Lastly, 3 participants highly emphasised with the statement that the incubators must know about the changing market needs to provide clear guidance to the students.

#### 6.6.6 Challenges of incubation centre

Identifying the major challenges with respect to incubation centre was a very important component of the study, where the students gave the following view - “Convincing the students on the importance of startup ….” (P1), “Students may not continue or misuse the opportunity ….” (P2), “Funds, resources, and lack of awareness among students ….” (P3; P4; P5; P6; P7; P8; P9; P10; P11; P12). According to the students, a major challenge faced by the incubation centre includes convincing the students to implement their own business ideas. Secondly, students state that some students may not be willing to continue their startups and may misuse the opportunities. However, most of the participants strongly argued that lack of funds, resources, and awareness among the students are the major challenges.

#### 6.6.7 Successful incubation centre

According to the students, the incubation centres can be successful with the following measures - “Certificate courses” (P1), “Focusing more on students ….” (P2; P3; P4; P5; P6; P7; P8; P9), “Advertisements, subsidies, exemption from taxation” (P2; P4), “Critically analysing various ideas, selecting the best ideas”(P2), “Encouraging students” (P3; P10; P11; P12). The incubation centre shall provide various certificate courses to the students and must highly focus on the student's capabilities. Further, they should go with aggressive advertisements, and subsidies and must provide an exemption from taxation for the highly efficient and feasible business plan. Interestingly, one of the participants recommended identifying the best business proposal from many alternatives. Students also stated that they need high encouragement to become a part of it. In this regard, one of the participants stated “Institutions must take a step forward to call the capable person to motivate them on this” (P1).

Considering the results of qualitative and quantitative analyses, an integrated analysis has been conducted and is presented in Table 3.

**Table 3:** Integrated analysis of quantitative and qualitative results.

Themes	Quantitative analysis	Qualitative Analysis	Integrated Analysis
**Knowledge and Awareness about the Incubation Centre**	They indicated a high level of awareness of the collective working environment & product development in the incubation centre encouraging more business ideas through brainstorming (M = 4.2424, SD = 0.75427).	They are aware that this is the platform to start a new business in their specialized field where their ideas can be checked for feasibility. Most of them became aware of the incubation centre after joining the master's course.	Students' knowledge and awareness of the incubation centre showed a similar finding between qualitative and quantitative results. Additionally, the qualitative results revealed the significance of the Masters's course in gaining insights into the incubation centre.
**Perception about Incubation Centre**	Students highly perceived that the reputation and credibility (M = 4.0000, SD = 0.84194) of the incubation centre and values of the social circle (M = 3.7778, SD = 0.92838) are very vital.	Most of them stated that incubation centres are meant to provide guidance, support, and motivation for their business ideas.	The perception was found to be contrary as quantitative results showed the relevance of reputation, credibility, and social circle in the incubation centre. Whereas qualitative results revealed the importance of guidance, support, and motivation for their business ideas.
**Education and Collaboration**	Building a strong network is an essential part of the learning experience from incubation centre (M = 4.2828, SD = 0.78001).	It provides a platform to transform their ideas into actual reality and also helps them to get funding. They also recommend institutions take a step forward to collaborate with the incubation centres, companies, investors, etc.	Here, the quantitative results are in line with the qualitative results ensuring the pivotal role of collaborations.
**Policy and Regulatory Framework**	Students highly agreed that clear government policies attract investments in incubator programs (M = 4.0707, SD = 0.84497). Students also believe that the incubation centre encourages experimentation and flexibility (M = 4.1515, SD = 0.75793) and fosters entrepreneurship (M = 4.1515, SD = 0.77117).	Here, the students stated the certification of incubation centre, at the same time they stated the importance of Government support in enhancing incubation centre and encouraging new businesses.	Similarly, the views on the policy and regulatory framework were found to be similar which pressurised the role of Government support.
**Role of Incubators**	Students reported that ongoing monitoring and feedback loops contribute to continuous improvement and growth among startups (M = 4.1313 SD = 0.80059).	Students reported that the incubators must visit their institutions and promote their centre. Moreover, they shall guide the students with their ideas. Participants highly emphasised the statement that the incubators must know about the changing market needs to provide clear guidance to the students.	Students' views on the role of incubators were found to be in line with each other highlighting their guidance and monitoring.
**Challenges of Incubation Centre**	They stated that incubation centres are facing a challenge in identifying startups with high growth potential (M = 3.9697, SD = 0.89391).	Major challenges faced by the incubation centre include convincing the students to implement their own business ideas. Secondly, students state that some students may not be willing to continue their startups and may misuse the opportunities. However, most of the participants strongly argued that lack of funds, resources, and awareness among the students are the major challenges.	The results here indicated a supportive finding which showed identifying the potential and convincing the students to accelerate entrepreneurship.
**Successful Incubation Centre**	Engaging alumni and successful graduates as mentors and advisors in the incubation centre (M = 4.1212, SD = 0.82090).	They should go with aggressive advertisements, and subsidies and must provide an exemption from taxation for the highly efficient and feasible business plan. Interestingly, one of the participants recommended identifying the best business proposal from many alternatives. Students also state that they need high encouragement to become a part of it. With this regard, one of the participants stated “Institutions must take a step forward to call the capable person to motivate them for this” (P1).	This finding gave a larger notion on the promotion of incubation as well as aggressive attempts of Management Institutes to conduct the awareness campaign by inviting capable personnel to boost students' ideas.

Overall, education along with collaboration acts as a major catalyst to enhance the effectiveness of the incubation centre with r exceeding 0.5 (P < 0.01**). Furthermore, students’ perception, education collaboration, and policy regulatory framework highly impacted the incubation centre, which further enhanced its success according to the students. When there are low level of challenges, the role of the incubation centre on its success is more but moderate (β = 0.743, P < 0.001**), and high level of challenges (β = 0.567, P < 0.001**) reduce the influence of the incubation centre on its success. These results indicate the significant role of incubation centres in accelerating the development of entrepreneurship. At the same time, it also proves that incubation centres are effective and successful according to students, as they have stated favourable view on perception, policy and regulatory framework, collaboration and education within the incubation centre.

## 7 Discussion

Incubators serve as a critical role in transforming academic research into sustainable commercial businesses. They create a revenue-generating environment by developing strong links between institutions, business sponsors, government bodies, and society [65]. In this regard, this study aimed to provide a comprehensive understanding of incubators' role in accelerating the development of entrepreneurship and also to explore the effectiveness and success of incubators through various indicators, by taking students' views. The results provided a comprehensive picture of students' views on the incubation centre from quantitative as well as qualitative research perspectives. As per the qualitative research perspective, the students reported that, their awareness about incubation setups is connected to their participation in the masters' degree, indicating that educational initiatives on dissemination of information about entrepreneurship. This indicates the role of management institutes to take initiative, which is highly significant in altering students' involvement in these centres to embark on entrepreneurial journey. Students are aware that workshops and camps, training programs, collective working environment & product development through incubation centres enhance more business ideas, enabling brainstorming, as [66] stated that starting a business is a result of innovation. The study also found that the reputation and credibility of the incubation centre and building a strong network is an essential part of learning from it, supported by Ref. [65], and has argued persuasively towards the importance of networking in providing start-ups with resources, competencies, expertise, learning, and social capital. The students have also agreed with the clear government policies, helping them to attract investments in incubator programs. They have identified the importance of taxation policies and industrial regulations that ease financial burdens on startups. Furthermore, a well-structured incubator layout and resources, ongoing monitoring, and feedback loops were recommended by the students. However, students stated, initial market entry barriers and competition are the major challenges for them; providing a conducive and innovative environment for startups is a challenge for the Management Institutes and identifying startups with high growth potential is another major challenge for the incubation centre. Hence, it is argued that the collaboration among educational institutions, investors, financiers, and business professionals is vital [67,68]. It was interesting to note that Education along with collaboration is the major catalyst to enhance the effectiveness of the incubation centre. These findings support the previous studies, which report that incubation centres initially provide incubated start-ups with a large network and increased credibility [65,69]. Following that, mentors and coaches provide specialized knowledge, capabilities, guidance, and inspiration, while also promote relationships and aid in giving targeted recommendations [70,71]. Finally, external players inside the network of the incubation centre provide funding, technical experience, and market insights to start-ups [72]. However, there was no significant variation among the gender and course of the students with regard to their view of the incubator centre, which indicates equal inclusivity between males and females as well as commerce and management streams. Overall, the study found that students’ perception, education collaboration, and policy regulatory framework pertaining to incubation centres highly impacted the effectiveness of the incubation centre, which further enhanced its success. In this regard, the system of education should foster an atmosphere that allows young people to transition from employees to entrepreneurs, while also prepare them to enhance their skills and knowledge in order to generate jobs [4]. However, awareness of the incubation centre showed no impact on the role of the incubation centre. Furthermore, it was revealed, when there is low level of challenges, the role of the incubation centre on its success is more moderate and high level of challenges reduce the influence of the incubation centre on its success. Hence, extensive approaches must be undertaken to tackle these challenges with collaborative efforts from the incubation centre.

## 8 Conclusion

Present research examined the role of incubation centre in accelerating entrepreneurship among university students by giving special focus towards student's awareness, perception, education along with collaboration, policy and regulatory framework and its relevance in enhancing the effectiveness of incubation centre leading to its success. These objectives were fulfilled through mixed method approach using social capital theory and institutional theory which revealed that education along with collaboration acts as a major catalyst to enhance the effectiveness of the incubation centre compared to mere students' perception, awareness, and policy regulatory framework. Moreover, the study reported that when there are low level of challenges, the role of the incubation centre on its success is more. These results indicate the significant role of incubation centres in accelerating the development of entrepreneurship. At the same time, it also proves that incubation centres are effective and successful according to students, as they have stated favourable view on perception, policy and regulatory framework, collaboration and education within the incubation centre.

The existing literature provides a deeper understanding and the results highlights the importance of incubation centres. Business incubators, which are the key boosters of entrepreneurship, give young entrepreneurs the essential instruments, sources, and professional direction they need to successfully make the difficult transition from an idea to a successful business and the positive effects which a well-designed incubation program may have on the local start-up ecosystem. Personalized resources, mentoring, and network access have fuelled entrepreneurs in a variety of industries, supporting regional economic growth. It is also expected to take informed decisions as well as to meet the essential requirements by overcoming the potential barriers. For the success of businesses by entrepreneurs, these kinds of support are expected from the institutions. Moreover, financial resources as well as legal assistance are required to access the market data to meet the complexities of the business. For driving sustainable development, it is important to bring the collaborative ecosystem that promotes exchange of ideas and knowledge among mentors, investors, government bodies, industries, entrepreneurs, and other stake holders. Practically, the prosperous businesses that emerged out of these incubators demonstrate how effective they are in fostering quick development, job creation, and industry innovation. However, a thorough ecosystem involving physical workplaces, access to knowledgeable mentors, exposure to market data, access to financial and legal assistance, and prospective investment opportunities all together form an all-encompassing environment that may help entrepreneurs succeed. These successes offer powerful inspiration, reiterating the strong case for boosting international investment and expanding company incubation efforts.

### 8.1 Implications to theory and practice

#### 8.1.1 Implication to theory

As far as the theoretical implication of this study is concerned, we have explored some new constructs applicable to the studies pertaining to incubation centre and its effectiveness. Moreover, this study was the first one to implement mixed method approach and provided an extensive overview of incubation centre in accelerating entrepreneurship development from the perspective of students. First and foremost, there is a scarcity of tested research on the role of incubation centres in entrepreneurship development among students, considering which present study has explored qualitative and quantitative outcome which provided the answers to more comprehensive questions pertaining to incubation centre as quantitative approach proved that students' perception, education along with collaboration, policy and regulatory framework are vital for incubation success. Also, the qualitative research provided more comprehensive understanding on the role of management institutes in spreading awareness, seeking guidance and infrastructure. Particularly, present study was grounded on the social capital theory and institutional theory that provide a complete understanding of the factors that are included in the success of the incubation centres and that foster the student's interest towards entrepreneurship. Additionally, these theories provided the framework to assess students' perception on structure, facilities offered, place, and mentoring in order to support the entrepreneurship. Moreover, the institutional theory underscores the policy and regulatory framework in shaping the organisational and individual behaviour. There are significantly few research which used these two theories to understand the incubation centre in accelerating the entrepreneurship development. Apart from the existing concepts indicated in the existing theories, present study has made a significant theoretical contribution to the existing literature by identifying the relevance of education along with collaboration, investor networking, and network building in enhancing students' involvement in incubation centres for accelerating entrepreneurship development. Further, it explains the collaboration arrangements made by the educational institutions with incubation centres to achieve the sustainable development and support innovative approaches to enhance the value of participation in an incubator.

#### 8.1.2 Practical implications

Considering the significant gap in the present domain, this study was carried out for an upcoming business generator with the innovative ideas. The qualitative results of the present study revealed the significance of the Masters's course in gaining insights into the incubation centre. Moreover, the study reports that the major challenges faced by the incubation centre include convincing the students to implement their own business ideas which can further be boosted through the institutions and centres by organising orientation programmes, seminars, and marketing initiatives to introduce the idea of incubation centres in the curriculum, and this can help to promote entrepreneurship development. Moreover, quantitative results showed the relevance of reputation, credibility, and social circle in the incubation centre and qualitative results revealed the importance of guidance, support, and motivation for their business ideas. This can be tackled and implemented through building a strong network which is an essential part of the learning experience from incubation centre that states the prominence of educational institutions' collaboration between the local and regional businesses, government bodies, financial institutions, venture capitalists with the entrepreneurs, and key stakeholders. Moreover, as per the outcome, students perceive that it provides a platform to transform their ideas into actual reality and also help them to get funding, where an initiative can be undertaken by the incubation centre to demonstrate how the ideas are practically put into practices. Administrators and managers can capitalise on this opportunity to build a network to improve the learning abilities of young entrepreneurs within the incubation setting and the reach of incubation programs. As the study found the significance of education along with collaboration in enhancing the effectiveness of incubation centre, more emphasis should be given on institutions providing support services through collaboration in terms of funding sources, access to resources, mentoring, and monitoring, as proved in the study. This collaborative service will contribute to continuous improvement and growth among startups as well as help to understand the industry dynamics and best practices. Lastly, students highly agreed that clear government policies attract investments in incubator programs and also stated the certification of incubation centre, at the same time they stated the importance of Government support in enhancing incubation centre and encouraging new businesses. As a result, the study recommends that there is a need for policy and regulatory frameworks to ensure smooth progress in the operations of the incubation centres. This argues the intervention of Government in boosting entrepreneurship, which can be inculcated in one of the policies and guidelines formulated to promote Micro Small and Medium Enterprises sectors in India. The findings of the study can be roadmap for Government bodies to provide financial assistance, infrastructure, and regulatory support as they facilitate ease of doing business through incubator networks. Policy makers ought to give priority to entrepreneurship programmes that enhance constant innovation within competitive startup ecosystem.

#### 8.1.3 Managerial implications

This study also provides an in depth understanding of the perceptions of the students about the incubation centres among the administrators. This study assists the decision-makers and administrators of educational institutions to set up or aspire to establish incubation centres. In order to address the problems faced by the students, including resources, lack of funds, and awareness, the educational institutions need to focus on providing proper guidance, modern amenities, effective testimonials of successful incubation centres, an alumni network, and adopting different strategies based on changing market dynamics. For the effective implementation of incubations through the collaboration of educational institutions with various industries as well as industrial experts through well-designed curriculum, networking events, mentorship, workshops, and orientation programs to encourage and boost the knowledge of the students at the grass-root level. Finally, administrators tend to take a proactive review system for the ongoing improvement of incubation centres. Outcome of regular evaluations, comments of the stakeholders, and market trends may help influence strategic decision-making and keep incubation centres adaptable to changing requirements and possibilities.

### 8.2 Key lessons learnt

Numerous key elements from the qualitative and quantitative approach which will be crucial to expand the scope of incubation centres have been identified. The results of the study highly emphasised that the incubators must know about the changing market needs to provide clear guidance to the students. It was also stated that incubation centres are facing a challenge in identifying startups with high growth potential, where the major challenges faced by them include convincing the students to implement their own business ideas. Secondly, students state that some students may not be willing to continue their startups and may misuse the opportunities. Moreover, engaging alumni and successful graduates as mentors and advisors in the incubation centre can encourage the budding entrepreneurs in management institutes. Incubators should go with aggressive advertisements, and must provide subsidies and an exemption from taxation for the highly efficient and feasible business plan. Interestingly, one of the participants recommended identifying the best business proposal from many alternatives will contribute to its success. They also state that they need high encouragement to become a part of it, where institutions must take a step forward to call the capable person to motivate them for this.

### 8.3 limitations of this research

Further, the present study's domain is limited only to the students and data was collected only from 3 universities of Dakshina Kannada district. This will restrict the generalisation of the study findings to other populations. The qualitative method of collecting data relied on only in-depth interviews for small number of samples through purposive sampling method, which may not provide overall opinion of the students because these are self-reported leads to inaccuracies and prejudices.

### 8.4 Future research recommendations

As this study restricted its scope to understand the students' perspectives from Management institute, future studies can highly ponder on taking all the stakeholders' perspectives with 360-degree approach with mixed methodology. Here, the challenges and realistic perspective from incubation centres, academicians, Government authorities, industries and students would strongly contribute to the improvement of incubation centre in all the spheres. Further, this study only focused on management institutes, future research can ponder more on engineering and medical institutes which is an integral and vital part of future entrepreneurship trends. Future studies can also explore the effectiveness of incubation centres with multidisciplinary budding entrepreneurs. Apart from management capabilities, expertise in product development, design, medical needs, AI, Big Data, etc., can contribute in formulating the best business proposals. The findings of the study establish a strong base for further investigation into the potential of business incubators in different directions. Further, exploration on the experiences of the students, inspiration factors within the incubation centres, and perceptions of the students to understand the relationship between organisational, individual, and environmental aspects which influence the entrepreneurship education and startup development through qualitative research. To measure the specific dynamics of the incubation centres, separate exploratory research is recommended, focusing on alumni engagement tactics, the efficacy of mentorship and monitoring, and the role of the academic and cultural framework in influencing the success of the program. A comparative study among various types of incubation models, incubation centres, and different geographical areas will help in addressing the particular challenges and needs of entrepreneurs in diverse contexts. On the basis of an additional examination on the effectiveness of the incubation centres programs, it is suggested to conduct longitudinal studies to evaluate the long-term impact. Future investigations could focus on areas such as evaluating the enduring impact of start-ups post-incubation, elucidating incubators’ role in driving technological innovation, aligning incubators with sustainability objectives, harnessing the power of digital platforms for incubator enhancement, and adapting to the ever-evolving entrepreneurial landscapes. Future research can also concentrate more on assessing the incubation centre practices in developed countries to implement the feasible and successful practices in developing countries. Continual research in these domains promises to refine our understanding and boost the efficacy of business incubators as vital catalysts for entrepreneurship and economic advancement.

## Funding

Open access funding is provided by Yenepoya(Deemed to be University), Karnataka, India.

## Additional information

No additional information is available for this paper.

## Data availability statement

The data supporting this study's findings are not publicly available due to confidentiality and other ethical considerations. However, data may be available from the authors upon reasonable request and with permission from the institution.

## CRediT authorship contribution statement

**Niyaz Panakaje:** Writing – original draft, Validation, Supervision, Methodology, Formal analysis. **Arun A. Bhagwath:** Writing – original draft, Supervision, Conceptualization. **S.M. Riha Parvin:** Writing – review & editing, Methodology, Data curation, Conceptualization. **Madhura K:** Data curation. **Ujwala Kambali:** Conceptualization.

## Declaration of competing interest

The authors declare that they have no known competing financial interests or personal relationships that could have appeared to influence the work reported in this paper. All the authors herby declare no competing interest.
