# Supplementary material for: Accelerating entrepreneurship: Evidence from the incubation centers of management institutes of Dakshina Kannada
Source: Heliyon. 2024 Jul 9;10(14):e34312. doi: 10.1016/j.heliyon.2024.e34312 (PMC11301167; doi:10.1016/j.heliyon.2024.e34312)
Supplement: Multimedia component 2 [file mmc2.docx]

**UNDERSTANDING INCUBATORS AS A TOOL FOR ACCELERATING ENTREPRENEURSHIP: EVIDENCE FROM MANAGEMENT INSTITUTE OF DAKSHINA KANNADA**

***QUESTIONNAIRE***

|  | Name |  | | | | | |
| --- | --- | --- | --- | --- | --- | --- | --- |
|  | Age |  | | | | | |
|  | Gender | Male | | | Female | | |
|  | Course | Under Graduation | | | Post Graduation | | |
|  | Stream of Education | Commerce | | Management | | | |
|  | Specialization | HR | Finance | | | | Income Tax |
|  |  | Insurance and Banking | | | | Entrepreneurship | |
|  |  |  | |  | | | |

| **Sl. No** |  |  | |
| --- | --- | --- | --- |
| 1 | Have you studied entrepreneurship as a subject in your course? | Yes | No |
| 2 | Does your family run any business? | Yes | No |
| 3 | Do you have any idea of an incubation center? | Yes | No |

**Awareness:**

| **Sl. No** | **Statements** | **Strongly Agree** | **Agree** | **Neutral** | **Disagree** | **Strongly Disagree** |
| --- | --- | --- | --- | --- | --- | --- |
| 1. **Workshops and Camps** | | | | | | |
|  | It helps to learn more about incubators. |  |  |  |  |  |
|  | It helps to understand how business incubators support startups. |  |  |  |  |  |
|  | It shows real-life examples of successful startups and their incubator involvement. |  |  |  |  |  |
| 1. **Collective working environment** | | | | | | |
|  | It increased my awareness of the benefits of working in a shared space with other startups. |  |  |  |  |  |
|  | The collaborative setting can foster innovative thinking and idea exchange. |  |  |  |  |  |
|  | It encourages the more business ideas through brainstorming. |  |  |  |  |  |
| 1. **Training Programs** | | | | | | |
|  | It effectively explains the role of business incubators in supporting startups. |  |  |  |  |  |
|  | The content covered enhanced my understanding of how incubators can accelerate entrepreneurship. |  |  |  |  |  |
|  | It improved my knowledge of the benefits that business incubators. |  |  |  |  |  |

**Perception:**

| **Sl. No** | **Statements** | **Strongly Agree** | **Agree** | **Neutral** | **Disagree** | **Strongly Disagree** |
| --- | --- | --- | --- | --- | --- | --- |
| 1. **Reputation and Credibility** | | | | | | |
| 1 | It influences my confidence in the quality of services provided. |  |  |  |  |  |
| 2 | It helps to earn my trust in incubation centers. |  |  |  |  |  |
| 3 | Credibility is a key factor that influences my decision to engage with an organization. |  |  |  |  |  |
| 4 | I consider an incubator's history and track record when evaluating incubation centers. |  |  |  |  |  |
| 5 | The success stories of firms that have graduated from the incubator enhance its credibility. |  |  |  |  |  |
| 6 | The incubator's alumni network influences my perception |  |  |  |  |  |
| 1. **Cultural and Social Influence** | | | | | | |
| 1 | My cultural background and the Role and purpose of the incubation centers. |  |  |  |  |  |
| 2 | My social group evaluates the effectiveness of incubation centers in nurturing startups. |  |  |  |  |  |
| 3 | The beliefs within my community influence my perception of the resources provided by incubation centers. |  |  |  |  |  |
| 4 | The values of my social circle judge the impact of incubation centres on fostering innovation and entrepreneurship. |  |  |  |  |  |

**Education Along with Collaboration:**

| **Sl. No** | **Statements** | **Strongly Agree** | **Agree** | **Neutral** | **Disagree** | **Strongly Disagree** |
| --- | --- | --- | --- | --- | --- | --- |
| 1. **Investor Networking** | | | | | | |
| 1 | It enriches the educational experience of startup incubation programs. |  |  |  |  |  |
| 2 | Collaborative interactions with investors contribute to a more comprehensive understanding of entrepreneurial concepts. |  |  |  |  |  |
| 3 | Investor networking contributes to a more practical and applied understanding of entrepreneurship. |  |  |  |  |  |
| 4 | Networking opportunities with investors enhance my learning experience within the incubation setting. |  |  |  |  |  |
| 1. **Market Accessibility** | | | | | | |
| 1 | Having access to real market insights enhances the educational value of startup incubation programs. |  |  |  |  |  |
| 2 | industry experts provide a better understanding of how entrepreneurial concepts apply to the market. |  |  |  |  |  |
| **3** | Learning about market dynamics gives me a more practical understanding. |  |  |  |  |  |
| 1. **Network Building** | | | | | | |
| 1 | Building a strong network is an essential part of my learning experience in a startup. |  |  |  |  |  |
| 2 | The different individuals and groups enrich my educational experience. |  |  |  |  |  |
| 3 | Developing connections with professionals from different fields improves my learning within the incubation setting. |  |  |  |  |  |

**Policy And Regulatory Framework:**

| **Sl. No** | **Statements** | **Strongly Agree** | **Agree** | **Neutral** | **Disagree** | **Strongly Disagree** |
| --- | --- | --- | --- | --- | --- | --- |
| - **Government policies** | | | | | | |
| 1 | An enabling policy environment encourages more startups to engage with incubators. |  |  |  |  |  |
| 2 | Clear government policies help to the attraction of investments in incubator programs. |  |  |  |  |  |
| 3 | Favourable government policies contribute to the ease of doing business through incubator networks. |  |  |  |  |  |
| 4 | The existence of supportive policies contributes to the decision to engage with incubator resources. |  |  |  |  |  |
| - **Taxation and incentives** | | | | | | |
| 1 | Taxation policies that favour startups in their growth within the incubation ecosystem. |  |  |  |  |  |
| 2 | Incentives offered by government policies make the appeal of participating in incubator programs. |  |  |  |  |  |
| 3 | Taxation policies that ease financial burdens on startups. |  |  |  |  |  |
| - **Industry regulations** | | | | | | |
| 1 | It encourages experimentation and flexibility. |  |  |  |  |  |
| 2 | It supports innovative approaches to enhance the value of participating in an incubator. |  |  |  |  |  |
| 3 | It fosters entrepreneurship and contributes to the effectiveness of incubators in nurturing startups. |  |  |  |  |  |

**Incubation Centres:**

| **Sl. No** | **Statements** | **Strongly Agree** | **Agree** | **Neutral** | **Disagree** | **Strongly Disagree** |
| --- | --- | --- | --- | --- | --- | --- |
| 1. **Structure and Facilities** | | | | | | |
| 1 | A well-designed incubator space enhances the environment for innovative thinking. |  |  |  |  |  |
| 2 | Access to modern amenities contributes to the value of the incubation experiences. |  |  |  |  |  |
| 3 | A well-structured incubator layout and resources create an inspiring setting for startups to thrive. |  |  |  |  |  |
| 4 | It helps to bring out scientific exploration and experimentation through Labs |  |  |  |  |  |
| 1. **Support Services** | | | | | | |
| 1 | It has potential links to funding sources. |  |  |  |  |  |
| 2 | It provides access to compliance services from professionals such as accountants and lawyers. |  |  |  |  |  |
| 3 | The capital from angel investors, government organizations, economic development coalitions, venture capitalists, and others encourages entrepreneurs. |  |  |  |  |  |
| 4 | The expert technology guidance fosters a more innovative and cutting-edge approach among startups. |  |  |  |  |  |
| 5 | It helps to get a tech-savvy and competitive startup ecosystem. |  |  |  |  |  |
| 6 | Proficient technology assistance provided by the incubator enriches the quality of entrepreneurial education. |  |  |  |  |  |
| 7 | Robust technology culture leads to continuous innovation within the incubation environment. |  |  |  |  |  |
| 1. **Mentoring and Monitoring** | | | | | | |
| 1 | Regular sessions on startups lead to strategic thinking and decision-making capabilities. |  |  |  |  |  |
| 2 | It helps to adjust strategies based on changing market dynamics |  |  |  |  |  |
| 3 | It helps to achieve personal and professional growth as entrepreneur. |  |  |  |  |  |
| 4 | It fosters a deeper understanding of industry dynamics and best practices. |  |  |  |  |  |
| 5 | Ongoing monitoring and feedback loops contribute to continuous improvement and growth among startups. |  |  |  |  |  |

**Challenges**

| **Sl. No** | **Statements** | **Strongly Agree** | **Agree** | **Neutral** | **Disagree** | **Strongly Disagree** |
| --- | --- | --- | --- | --- | --- | --- |
| 1. **Challenges of the Students** | | | | | | |
| 1 | Balancing academic commitments with entrepreneurial pursuits. |  |  |  |  |  |
| 2 | Lack of real-world business experience and practical skills. |  |  |  |  |  |
| 3 | Fear of failure and taking calculated risks. |  |  |  |  |  |
| 4 | Developing a strong and scalable business model. |  |  |  |  |  |
| 5 | Initial market entry barriers and competition. |  |  |  |  |  |
| 1. **Challenges of the Management Institutions** | | | | | | |
| 1 | Difficulty in adapting curriculum to startup needs. |  |  |  |  |  |
| 2 | Aligning with diverse startup goals. |  |  |  |  |  |
| 3 | Measuring program impact effectively. |  |  |  |  |  |
| 4 | Difficulty in finding industry connections. |  |  |  |  |  |
| 5 | Fostering effective collaboration between startups and faculty. |  |  |  |  |  |
| 6 | Providing a conducive and innovative environment for startups. |  |  |  |  |  |
| 1. **Challenges of Incubation Centers** | | | | | | |
| 1 | Identifying startups with high growth potential. |  |  |  |  |  |
| 2 | Sustaining adequate funding for operations. |  |  |  |  |  |
| 3 | Fostering innovation-driven culture. |  |  |  |  |  |
| 4 | Adapting to legal and regulatory changes. |  |  |  |  |  |
| 5 | Navigating evolving market trends and tech. |  |  |  |  |  |
| 6 | Bridging gaps in skill and knowledge areas. |  |  |  |  |  |

**SUCCESS**

| **Sl. No** | **Statements** | **Strongly Agree** | **Agree** | **Neutral** | **Disagree** | **Strongly Disagree** |
| --- | --- | --- | --- | --- | --- | --- |
| 1. **Innovation and Product development** | | | | | | |
| 1 | An incubator's shared resources can aid product development. |  |  |  |  |  |
| 2 | It provides resources for refining and developing new products |  |  |  |  |  |
| 3 | It fosters a competitive edge to the overall success of startups. |  |  |  |  |  |
| 1. **Skill development** | | | | | | |
| 1 | Enhanced skill development leads to improved problem-solving within startups. |  |  |  |  |  |
| 2 | It helps to seize the emerging opportunities. |  |  |  |  |  |
| 3 | This initiative within incubators creates a foundation for sustained growth and expansion of startups. |  |  |  |  |  |
| 4 | It fosters a culture of continuous learning, driving overall success. |  |  |  |  |  |
| 1. **Growth** | | | | | | |
| 1 | Through Strategic expansion of resources and partnerships |  |  |  |  |  |
| 2 | Through Continuous adaptation to evolving market needs and trends. |  |  |  |  |  |
| 3 | Scaling the capacity of incubation centres to accommodate more startups. |  |  |  |  |  |
| 4 | Engaging alumni and successful graduates as mentors and advisors. |  |  |  |  |  |
