# Supplementary material for: Accelerating entrepreneurship: Evidence from the incubation centers of management institutes of Dakshina Kannada
Source: Heliyon. 2024 Jul 9;10(14):e34312. doi: 10.1016/j.heliyon.2024.e34312 (PMC11301167; doi:10.1016/j.heliyon.2024.e34312)
Supplement: Multimedia component 1 [file mmc1.pdf]

YIASCM/PRN/447/2023 **25/05/2023**

Dear Dr. Niyaz Panakaje,

On behalf of the scientific research committee, I extend warm greetings to you.

This letter serves to confirm that the institutional ethical committee held its 3rd meeting on 25th May 2023 to review and discuss the application submitted via letter no. CBA/124/23 for conducting empirical research titled "*Understanding Incubators as A Tool for Accelerating Entrepreneurship: Evidence from Management Institute of Dakshina Kannada*" upon the request of the researcher. After careful consideration and observation of the questionnaire and related documents, the committee has issued the following decision.

Ethical committee Decision: Approved.

Title of the Proposed Study: "Understanding Incubators as A Tool for Accelerating Entrepreneurship: Evidence from Management Institute of Dakshina Kannada"

Date of Approval: 25th May 2023

Documents reviewed: Research Instrument/Questionnaire

Sincerely

Scientific Review Board

PRINCIPAL
The Yenepoya Institute of Arts, Science,
Commerce & Management
Balmatta, Mangaluru-575002
